# Neuronal hemoglobin in mitochondria is reduced by forming a complex with α-synuclein in aging monkey brains

**DOI:** 10.18632/oncotarget.7046

**Published:** 2016-01-27

**Authors:** Weiwei Yang, Xuran Li, Xin Li, Xuying Li, Shun Yu

**Affiliations:** ^1^ Department of Neurobiology, Xuanwu Hospital of Capital Medical University, Beijing, China; ^2^ Center of Parkinson's Disease, Beijing Institute for Brain Disorders, Beijing, China; ^3^ Beijing Key Laboratory for Parkinson's Disease, Beijing, China

**Keywords:** aging, hemoglobin, α-synuclein, mitochondrion, Parkinson's disease, Gerotarget

## Abstract

Neuronal hemoglobin (nHb) plays a critical role in maintaining normal mitochondrial functioning in the brain. However, in aging and Parkinson's disease (PD) brains, mitochondrial nHb levels are greatly reduced in neurons that accumulate α-synuclein (α-syn), suggesting a link between the two proteins. In this study, we demonstrate that α-syn and Hb can form a complex in both brain tissue and peripheral red blood cells (RBCs) in aging cynomolgus monkeys. nHb-α-syn complex levels in the mitochondrial fraction of the striatum decreased with age; this was negatively correlated with levels in the cytoplasmic fraction and in RBCs and was accompanied by a reduction in mitochondrial free nHb. In contrast, no changes in nHb-α-syn complex formation or free nHb levels were detected in the cerebellum. *In vitro* studies using a cultured dopaminergic cell line showed that intracellular accumulation of α-syn caused an elevation in nHb-α-syn complex levels in both mitochondrial and cytoplasmic fractions as well as a reduction in mitochondrial free nHb. nHb overexpression increased free nHb levels in mitochondria, stabilized mitochondrial membrane potential, and reduced α-syn-induced apoptosis. The above results suggest that α-syn forms a complex with nHb in selected regions of the aging brain, thereby decreasing mitochondrial function and increasing the risk of PD.

## INTRODUCTION

Aging brains are characterized by the formation of fibrous protein inclusions known as Lewy bodies (LBs) and Lewy neurites (LNs) [1, 2], which are associated with age-related neurodegenerative disorders such as Parkinson's disease (PD), PD dementia, and dementia with Lewy bodies [3, 4]. The major component of LBs and LNs is fibrous α-synuclein (α-syn), 90% of which is phosphorylated at serine 129 [5, 6]. α-Syn is a small acidic protein that is normally present in a soluble, monomeric form in neurons, particularly in presynaptic terminals and mitochondria [7, 8]. There is increasing evidence that formation of small α-syn aggregates or abnormal accumulation of the monomeric protein is detrimental to neurons [9]. While some studies indicate that α-syn oligomers are themselves toxic [10-12], others suggest that the pathogenicity of α-syn is linked to its interaction with other proteins [13-15], such as receptors, transporters and enzymes [16-20]; indeed, many proteins have been found to be colocalized with α-syn in LBs and LNs [21].

Nerve globins are a family of O_2_-binding proteins that include neuroglobin, cytoglobin, neuronal hemoglobin (nHb), and myoglobin [22]. These proteins are hypothesized to be involved in nervous system excitability, reactive nitrogen and oxygen species metabolism, and intracellular signaling pathways that regulate cell survival [22]. Among the most important physiological functions of these proteins is to sustain normal mitochondrial function [23-29]. Nerve globins have been implicated in the pathogenesis of neurodegenerative diseases. For example, nHb is abnormally expressed in PD brains [30]. nHb levels were reduced in dopaminergic neurons of the substantia nigra in PD patients, which also showed LBs or α-syn deposits [31]. Moreover, the ratio of mitochondrial to cytoplasmic nHb was found to be decreased in the substantia nigra of PD brains [30], which was associated with α-syn accumulation and impaired mitochondrial function [22, 32]. These findings imply that interaction between α-syn and nHb may impair mitochondrial function in PD brains.

Aging is the major risk factor for PD [33]. Increased α-syn accumulation and reduced mitochondrial Hb levels are also observed in aging brains, although these occur to a lesser degree than in PD brains [30]. Investigating the interaction between α-syn and Hb and its effect on mitochondrial function in aging brains may clarify the pathogenic mechanisms of PD and related diseases. We addressed this in the present study by comparative analysis of nHb-α-syn complex formation and its effects on mitochondrial function in the brains of cynomolgus monkeys of different ages. We also examined the association between age-dependent changes in Hb-α-syn complex formation in peripheral red blood cells (RBCs) to determine whether peripheral (p)Hb-α-syn complex levels reflect alterations in the central nervous system.

## RESULTS

### nHb-α-syn complex detection in monkey brain

**Figure 1:**
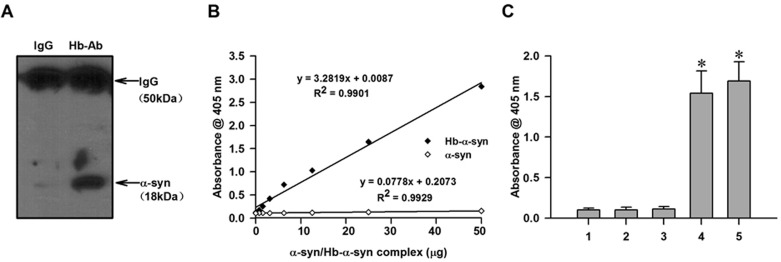
Demonstration of the presence of nHb-α-syn complex **A.** The amount of α-syn immunoprecipitated by a Hb antibody but not IgG was detected in striatum lysates. **B.** Hemoglobin beta chains (1 mg/ml) and recombinant α-syn (0.5 mg/ml) were incubated in phosphate-buffered saline (PBS) at 37°C for 24 h. Resultant Hb-α-syn complexes were separated by CO-IP and purified from the Protein G-Sepharose beads. Concentrations of purified Hb-α-syn complex and freshly prepared α-syn monomers were measured by enzyme-linked immunosorbent assay (ELISA) at an absorbance of 405 nm; absorbance was positively correlated with Hb-α-syn complex concentration, with an R^2^ value of 0.9901. There was no correlation between absorbance and α-syn monomer concentration. **C.** The ELISA method detected a clear signal in exogenous Hb-α-syn complex or a brain tissue sample, which was absent in the antibody and sample negative controls. Data are expressed as the mean ± SD. Tukey's multiple comparisons test after ANOVA, C: * *P* < 0.05, *vs*. negative control groups. Hb-Ab: hemoglobin antibody; 1: Hb antibody negative control; 2: Sample negative control; 3: Biotinylated 3D5 anti-α-syn mouse monoclonal antibody negative control; 4: ELISA for detecting exogenous Hb-α-syn complex; 5: ELISA for detecting brain tissue lysates.

The interaction between α-syn and nHb was examined by co-IP and western blotting. Brain extracts were precipitated with anti-Hb antibody and probed with an anti-α-syn antibody. An 18-kDa monomeric form of α-syn was detected in precipitates, suggesting that it interacts with nHb in the brain (Figure 1A). The presence of nHb-α-syn complex was also examined by the ELISA method specially designed to detect and quantify the nHb-α-syn complex. As was presented in the standard curve, the absorbance values were linearly correlated with the concentrations of Hb-α-syn complex, with a R^2^ value of 0.9901 (Figure 1B). The ELISA method detected a clear signal in a brain tissue sample, which was absent in the antibody and sample negative controls (Figure 1C). The ELISA result further confirmed the presence of nHb-α-syn complex in brain tissues and indicated that the established ELISA method could specifically detect and accurately quantify the Hb-α-syn complex.

### nHb and α-syn levels are altered in aging monkey brains

**Figure 2:**
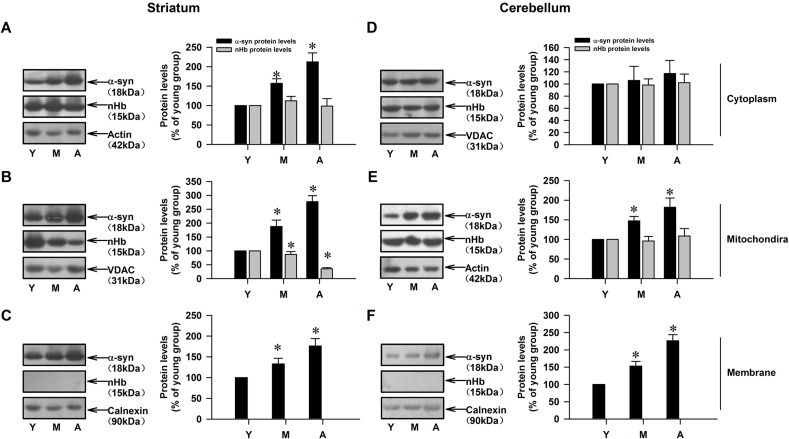
Age-dependent alterations of nHb and α-syn in cytosolic-, mitochondrial-, membrane fractions of monkey brains **A.** Levels of α-syn and nHb were detected in cytosol in striatum. **B.** Levels of α-syn and nHb were detected in mitochondria in striatum. **C.** Levels of α-syn and nHb were detected in membrane by Western blot in striatum. **D.**-**F.**. Levels of α-syn and nHb were detected in cytosol **D.**, mitochondria **E.**, membrane **F.** in cerebellum of monkey brain. Data are expressed as the mean ± SD. Tukey's multiple comparisons test after ANOVA, A/B/C: * *P* < 0.05, *vs*. young age group in the striatum (*n* = 4); D/E/F: * *P* < 0.05, *vs*. young age group in the cerebellum (*n* = 4); Y: young age group; M: middle age group; A: aged group.

We investigated the age-dependent changes in nHb and α-syn levels in different brain regions. In the striatum, an age-dependent increase in α-syn level was detected in all subcellular fractions, with the highest level observed in the mitochondrial fraction. nHb level did not change with age in the cytoplasmic fraction, but decreased in an age-dependent manner in the mitochondrial fraction (Figure 2A-2C). In the cerebellum, α-syn level increased with age in mitochondrial and membrane fractions but not in the cytoplasmic fraction, but there were no age-dependent changes in nHb in the cytoplasmic or mitochondrial fractions (Figure 2D-2F). nHb was absent in membrane fractions of the striatum and cerebellum (Figure 2C, 2F).

### nHb-α-syn complex levels are altered with age in different subcellular pools

**Figure 3:**
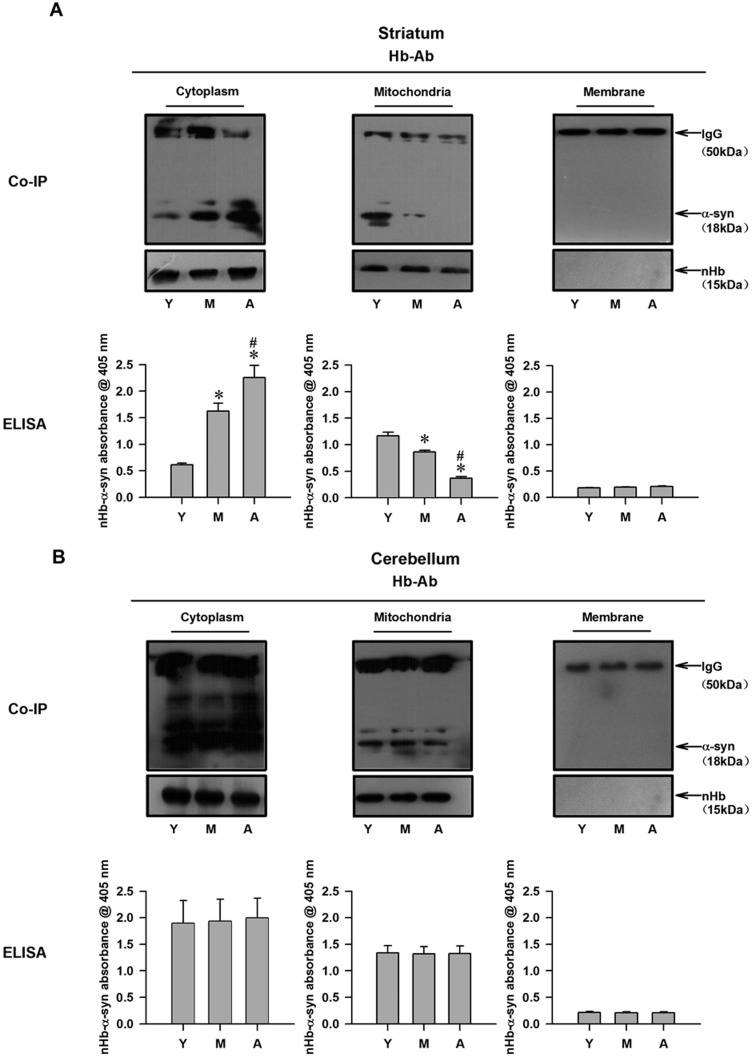
Detection of nHb-α-syn complex in cytosolic-, mitochondrial-, membrane fractions of monkey brains **A.** The amount of α-syn immunoprecipitated by a Hb antibody was changed in cytosolic- and mitochondrial-fraction in striatum with age, while the total amount of nHb expressed remained constant as indicated by Western blot analysis of the immunoprecipitated protein samples in all three fractions (upper panel). The same protein samples before IP were analyzed directly by ELISA. Levels of nHb-α-syn complex were detected in cytosol, mitochondria and membrane by ELISA (lower panel). **B.** The amount of α-syn immunoprecipitated by a Hb antibody was no change in cytosolic- and mitochondrial-fraction in cerebellum with age, and the total amount of nHb expressed remained constant in all three fractions (upper panel). The same protein samples before IP were analyzed directly by ELISA. Levels of nHb-α-syn complex were detected in cytosol, mitochondria and membrane by ELISA (lower panel). Data are expressed as the mean ± SD. Tukey's multiple comparisons test after ANOVA, A: *P < 0.05, *vs*. young age group in the striatum (*n* = 4). ^#^ *P* < 0.05, *vs*. middle age group in the striatum (*n* = 4). Hb-Ab: hemoglobin antibody; Y: young age group; M: middle age group; A: aged group.

Given that α-syn and nHb levels in the brain exhibit age-dependent changes, we speculated that nHb-α-syn complex levels are similarly altered by aging. In the striatum, nHb-α-syn complex levels in the cytoplasm increased in an age-dependent manner; this was inversely associated with the level in the mitochondrial fraction (Figure 3A). In the cerebellum, there were no age-dependent alterations in nHb-α-syn complex levels in either the cytoplasmic or mitochondrial fractions (Figure 3B). There was no nHb-α-syn complex detected in the membrane fractions of the striatum and cerebellum.

### pHb-α-syn complex levels are altered in RBCs

**Figure 4:**
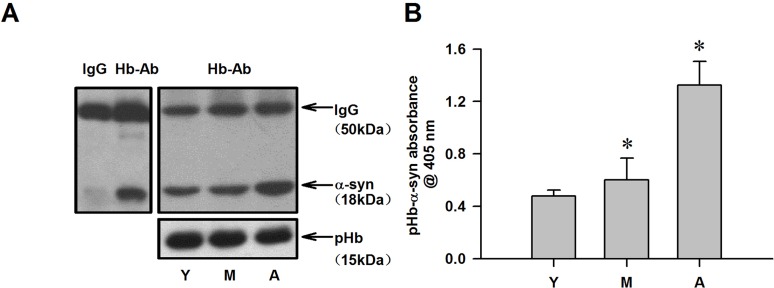
Detection of pHb-α-syn levels in RBC of monkeys **A.** The amount of α-syn immunoprecipitated by a Hb antibody was age-dependent increase in RBC lyses, while, the total amount of pHb expressed remained constant in three groups. **B.** pHb-α-syn levels were also detected using ELISA. Data are expressed as the mean ± SD. Tukey's multiple comparisons test after ANOVA, B: * *P* < 0.05, *vs*. young age group (*n* = 4). Hb-Ab: hemoglobin antibody; Y: young age group; M: middle age group; A: aged group.

α-Syn is present in plasma and passes through the cell membrane by passive diffusion. It is therefore possible that plasma α-syn enters RBCs and binds to pHb to form a pHb-α-syn complex. To determine whether a pHb-α-syn complex is present and to assess whether this is affected by age, RBCs from monkeys of different ages were lysed, and lysates containing mainly pHb were analyzed by Co-IP and ELISA. Lysates precipitated with an anti-Hb antibody and probed for α-syn revealed the presence of an pHb-α-syn complex in RBCs (Figure 4A). Moreover, RBC pHb-α-syn complex level increased with age, similar to age-dependent changes in cytoplasmic nHb-α-syn level in the striatum (Figure 4B).

### Correlation between brain nHb-α-syn and RBC pHb-α-syn complex levels

**Figure 5:**
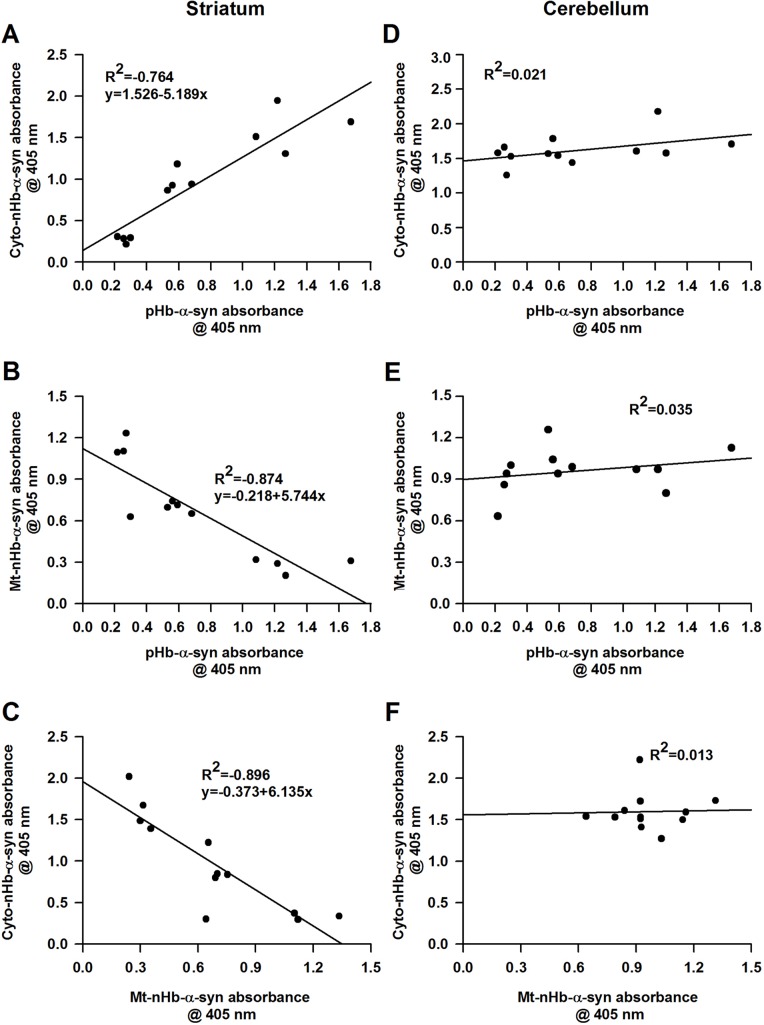
The relationship between cytosolic-, mitochondrial-, membrane-nHb-α-syn and pHb-α-syn **A.** Cyto-nHb-α-syn in striatum was positively correlated with pHb-α-syn, with an R^2^ value of 0.764 (*n* = 12, *P* < 0.05). **B.** Mt-nHb-α-syn in striatum was negatively correlated with pHb-α-syn, with an R^2^ value of 0.874 (*n* = 12, *P* < 0.05). **C.** Mt-nHb-α-syn in striatum was negatively correlated with cyto-nHb-α-syn, with an R^2^ value of 0.896 (*n* = 12, *P* < 0.05). **D.**-**E.** In cerebellum, the relationship between cyto-, mt-, and pHb-α-syn were detected using ELISA. **F.** The relationship between cyto- and mt-nHb-α-syn in cerebellum were detected using ELISA. Pearson correlation analysis was used to assess the association between nHb-α-syn in cyto-, mt-fractions and pHb-α-syn in RBC (*n* = 12). *P* < 0.05 was considered statistically significant. Cyto-nHb-α-syn: neuronal hemoglobin-α-syn complex in cytosolic fraction; Mt-nHb-α-syn: neuronal hemoglobin-α-syn complex in mitochondrial fraction; pHb--syn: peripheral hemoglobin-α-syn complex in RBC lyses.

We examined the correlation between striatal cytoplasmic and RBC Hb-α-syn complex levels and found that these were positively correlated (*n* = 12, R^2^ = 0.764, *P* < 0.05) (Figure 5A). In contrast, mitochondrial nHb-α-syn complex level in the striatum was negatively correlated with that in RBCs and in the cytoplasmic fraction (Figure 5B, 5C). There was no correlation between the pHb-α-syn complex level in RBCs and those of cytoplasmic (*n* = 12, R^2^ = 0.021, *P* > 0.05) or mitochondrial (*n* = 12, R^2^ = 0.035, *P* > 0.05) fractions of the cerebellum (Figure 5D, 5E). Meanwhile, the levels of nHb-α-syn complex in the mitochondria did not correlated with those of cytoplasmic (*n* = 12, R^2^ = 0.013, *P* > 0.05) fractions form the cerebellum (Figure 5F)

### nHb-α-syn complex formation reduces free mitochondrial nHb levels in cultured dopaminergic cells

**Figure 6:**
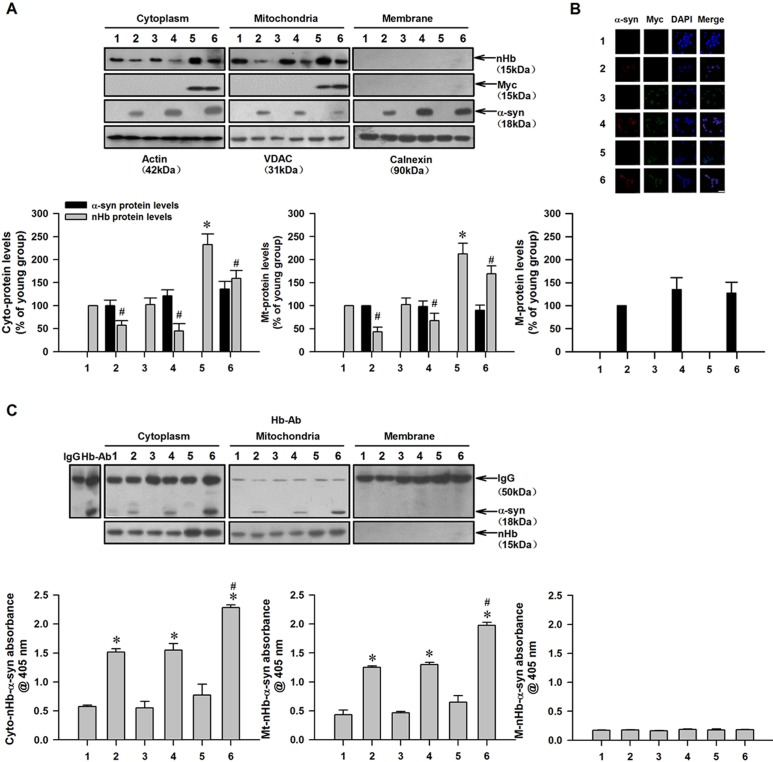
Reduction of free mitochondrial nHb levels by formation of nHb-α-syn complex in cultured dopaminergic cells **A.** The amount of free nHb, myc, α-syn expressed indicated by Western blot analysis of the protein samples before IP. Actin, VDAC, Calnexin were detected as a cytosol, mitochondria and membrane loading control, respectively. **B.** Cells were transfected with a myc or myc/nHb vector for 24 h with or without α-syn (10 μM) treatment for 6 h. At 24 h after transfection, MES23.5 cells fixed and stained with myc and α-syn antibody, followed by a mouse Alexa 488- or rabbit Alexa 594-conjugated secondary antibody. The nucleus was counterstained by DAPI. Bar = 50 μM. **C.** α-Syn immunoprecipitated by a Hb antibody was detected in cytosolic- and mitochondrial-fraction in 2, 4, 6 lanes (upper panel). Levels of nHb-α-syn complex were detected in cytosol, mitochondria and membrane by ELISA (lower panel). Data are expressed as the mean ± SD. Tukey's multiple comparisons test after ANOVA, A: * *P* < 0.05, *vs*. 1 group (*n* = 6); ^#^ *P* < 0.05, *vs*. 1, 3, 5 group, respectively (*n* = 6). B: * *P* < 0.05, *vs*. 1 group (*n* = 6); ^#^ *P* < 0.05, *vs*. 2 or 4 group (*n* = 6). Hb-Ab: hemoglobin antibody; Cyto-nHb-α-syn: neuronal hemoglobin-α-syn complex in cytosolic fraction; Mt-nHb--syn: neuronal hemoglobin-α-syn complex in mitochondrial fraction; M-nHb-α-syn: neuronal hemoglobin-α-syn complex in membrane fraction; 1: control group; 2: α-syn (10 μM) treatment for 6 h group; 3: myc vector transfection for 24 h group; 4: myc vector transfection for 24 h with α-syn (10 μM) treatment for 6 h group; 5: myc/nHb vector transfection for 24 h group; 6: myc/nHb vector transfection for 24 h with α-syn (10 μM) treatment for 6 h group.

Our results suggested that the formation of an nHb-α-syn complex reduced the pool of free nHb in mitochondria. To test this possibility, recombinant human α-syn was added to the culture medium of MES23.5 dopaminergic cells transfected with a vector expressing the human nHb gene or an empty vector and allowed to enter the cells by passive diffusion [8, 34]. There was no α-syn detected in untreated MES23.5 cells by western blotting or immunocytochemistry, suggesting that these cells have low levels of endogenous α-syn (Figure 6A, 6B). However, in α-syn-treated cells, strong immunoreactivity was detected in several subcellular fractions, including the cytoplasm, mitochondria, and plasma membrane (Figure 6A). MES23.5 cells expressed moderate levels of endogenous nHb in the cytoplasmic and mitochondrial fractions. In cells expressing human nHb, higher levels of nHb were detected in the cytoplasmic and mitochondrial fractions (Figure 6A). In all α-syn-treated cells, free nHb levels were lower in the mitochondrial and cytoplasmic fractions as compared to untreated cells (Figure 6A). nHb-α-syn complex formation and levels were increased in the cytoplasmic and mitochondrial fractions but not in the membrane fractions of α-syn-treated cells—especially in cells expressing human nHb—as determined by CO-IP and ELISA (Figure 6C).

### α-Syn overexpression reduces mitochondrial membrane potential (MMP)

**Figure 7:**
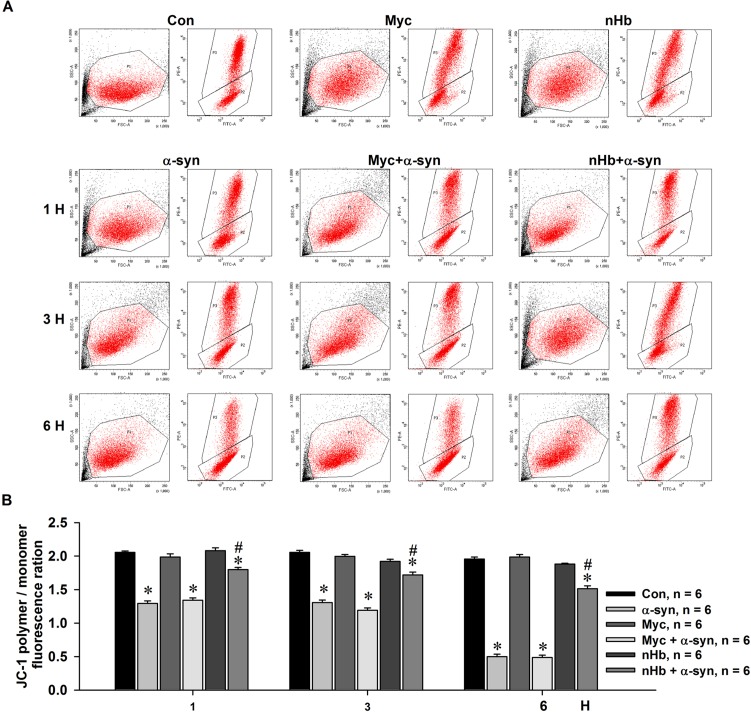
Effect of nHb on α-syn induced reduction of mitochondrial membrane potential MES 23.5 cells were transfected with a myc or myc/nHb vector for 24 h with or without α-syn (10 μM) treatment for indicated times. At 24 h after transfection, MES23.5 cells were stained by JC-1 and detected for the mitochondrial membrane potential. **A.** Representative images showed the J-aggregate (FL 2) and J-monomer (FL 1) fluorescence intensity. **B.** Statistical results showed the ratios of J-aggregate/J-monomer fluorescence intensity. Data are expressed as the mean ± SD. Tukey's multiple comparisons test after ANOVA, M: * *P* < 0.05, *vs*. control group (*n* = 6); ^#^ *P* < 0.05, *vs*. α-syn treatment groups (*n* = 6).

nHb stabilizes MMP; α-syn may bind to nHb and thereby reduce free nHb levels in mitochondria. To test this hypothesis, MES23.5 cells with or without nHb overexpression were treated with exogenous α-syn, which diffused into cells and resulted in intracellular α-syn accumulation. The MMP was then measured by JC-1 staining. MMP was markedly reduced in cells treated with α-syn. However, this decrease was partly abrogated in cells expressing human nHb as compared to control cells (Figure 7A, 7B). This was consistent with the observation that nHb-overexpressing cells had higher levels of free nHb in the mitochondria despite the formation of the nHb-α-syn complex.

### Attenuation of α-syn-induced apoptosis by nHb overexpression

**Figure 8:**
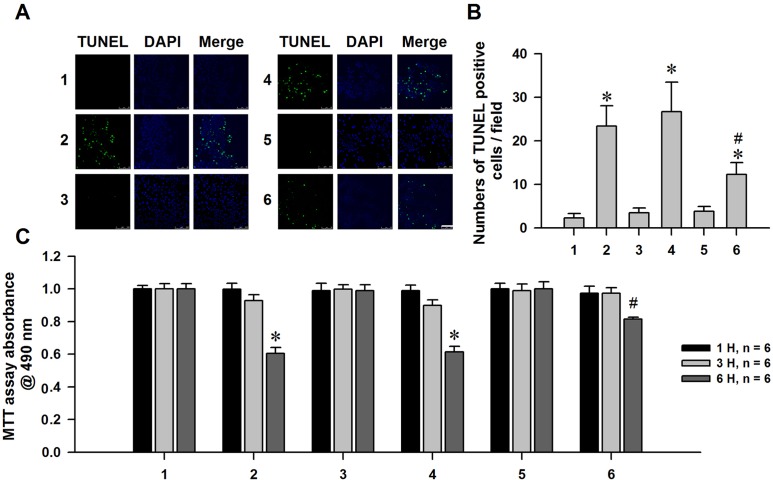
Neuroprotective effect of nHb against α-syn induced dopaminergic neuronal cells injuries **A.** Cells were transfected with a myc or myc/nHb vector for 24 h with or without α-syn (10 μM) treatment for 6 h. At 24 h after transfection, MES23.5 cells fixed and stained with TUNEL, the nucleus was counterstained by DAPI. Bar = 50 μM. **B.** Statistical results showing the TUNEL positive cells. C: MTT assay of the cell viability in 1 - 6 groups with or without addition of α-syn (10 μM) for 1, 3, 6 h. Data are expressed as the mean ± SD. Tukey's multiple comparisons test after ANOVA, B/C: * *P* < 0.05, *vs*. 1 group (*n* = 6); ^#^ *P* < 0.05, *vs*. 2 or 4 group (*n* = 6). 1: control group; 2: α-syn (10 μM) treatment for 1, 3, 6 h group; 3: myc vector transfection for 24 h group; 4: myc vector transfection for 24 h with α-syn (10 μM) treatment for 1, 3, 6 h group; 5: myc/nHb vector transfection for 24 h group; 6: myc/nHb vector transfection for 24 h with α-syn (10 μM) treatment for 1, 3, 6 h group.

A reduction in MMP has been shown to induce apoptosis [35]. Given that α-syn treatment decreased MMP in MES23.5 cells, we speculated that α-syn can induce apoptosis in these cells. Indeed, we found that α-syn treatment increased the number of TUNEL-positive cells and decreased cell viability. nHb overexpression in α-syn-treated cells suppressed apoptosis, suggesting that nHb has protective effects (Figure 8A-8C).

## DISCUSSION

The results of this study demonstrate that the Hb-α-syn complex is present in the brain and in peripheral RBCs, and that the levels change with age. This can explain previous observations that mitochondrial nHb is reduced in specific regions of the aging brain [30]. Indeed, in the striatum of aging cynomolgus monkeys, we found that nHb-α-syn complex levels in mitochondria decreased with age, which was negatively correlated with age-dependent increases in complex formation in the cytoplasm. Concomitant with changes in nHb-α-syncomplex levels, the concentration of free nHb in the mitochondrial fraction declined in an age-dependent manner, while α-syn accumulation was observed in cytoplasmic and mitochondrial fractions. In contrast, free nHb and nHb-α-syn complex levels did not change with age in cytoplasmic or mitochondrial fractions of the cerebellum. Based on these observations, we speculate that accumulation of cytoplasmic α-syn is the major factor contributing to the reduction in mitochondrial free nHb in aging brains.

nHb levels in mitochondria and cytoplasm are normally maintained in a dynamic equilibrium. Accumulation of α-syn in the cytoplasm leads to an increase in nHb-α-syn complex formation and reduces nHb translocation from the cytoplasm to mitochondria, thereby decreasing mitochondrial free nHb and nHb-α-syn complex levels. Although mitochondrial α-syn accumulation would also tend to increase nHb-α-syn complex formation and accordingly diminish the pool of free mitochondrial nHb, this is not likely to be the major reason for the age-dependent reduction in mitochondrial free nHb. This is supported by the observation that in the cerebellum, age-dependent α-syn accumulation in the mitochondrial fraction was not accompanied by a reduction in mitochondrial free nHb. Moreover, *in vitro* experiments using cultured MES23.5 dopaminergic cells showed that extracellular application of recombinant human α-syn induced a rapid elevation in α-syn levels and nHb-α-syn complex formation in cytoplasmic and mitochondrial fractions, which was inversely associated with a decrease in free mitochondrial nHb level. In contrast, α-syn accumulation in cultured MES23.5 cells led to an increase in nHb-α-syn complex formation in the mitochondrial fraction. The reason for the discrepancy is not clear, but one possibility is that the baseline concentration of nHb-α-syn complex in mitochondria is low in α-syn-untreated cells due to low expression of endogenous nHb-α-syn complex, and addition of exogenous α-syn induced a rapid increase in mitochondrial α-syn and thus, nHb-α-syn complex levels, which did not return to baseline within the observed time period. Nonetheless, our findings demonstrate that formation of a nHb-α-syn complex reduces the levels of free mitochondrial nHb.

Intracellular nHb preferentially localizes to mitochondria, where it plays a critical role in protecting against rotenone-induced mitochondrial injury [24]. Mitochondrial nHb has been shown to increase MMP, which plays a key role in ATP production [36]. We therefore examined whether the α-syn-induced decrease in mitochondrial nHb is associated with reduced MMP and increased apoptosis. Indeed, application of exogenous α-syn induced intracellular α-syn accumulation and reduced mitochondrial free nHb as well as MMP. However, the latter was inhibited in nHb-overexpressing cells, indicating the role of mitochondrial nHb in stabilizing the MMP. Given that a decrease in MMP is among the earliest events in mitochondria-mediated apoptosis [35], we examined whether the α-syn-induced reduction in MMP was associated with increased apoptosis, and found that the number of TUNEL-positive cells was increased while cell viability was reduced by α-syn treatment. This was mitigated by overexpressing nHb in α-syn-treated cells. These results suggest that nHb can protect against α-syn-induced apoptosis, possibly by stabilizing MMP.

Since peripheral RBCs also contain pHb and α-syn is present in the plasma and various cell types including RBCs [37], we verified whether α-syn is bound to pHb in RBCs. We detected the formation of an pHb-α-syn complex in RBCs and showed that the level increased with age; this was positively correlated with the level in the cytoplasmic fraction of the striatum. This suggests that changes in pHb-α-syn complex levels in the periphery reflect those in the brain, which is supported by the fact that neuronal α-syn is secreted into extracellular space, passes through the brain-blood barrier into plasma, and enters RBCs. However, additional studies are needed to determine the source of α-syn in RBCs.

## CONCLUSIONS

In the present study, we provide evidence for Hb-α-syn complex formation in the brain and in peripheral RBCs, and demonstrate that mitochondrial nHb level decreases with age in the striatum as a result of increased intracellular α-syn accumulation and nHb-α-syn complex formation. We also show a correlation between Hb-α-syn complex levels in the brain and in the periphery, and demonstrate that the reduction in mitochondrial nHb level contributes to α-syn-induced mitochondrial dysfunction and apoptosis. These results provide a basis for the preferential degeneration observed in certain brain regions such as the striatum in PD and in the aging brain.

## MATERIALS AND METHODS

### Plasmid constructs

Human wild-type (WT) nHb subunit beta (WT-nHbB, WT-nHb) cDNA was reverse transcribed from human brain RNA using the following forward and reverse primers for nHb: 5′-TCCACTCCTGATGCTGTTATG-3′ and 5′-CCAGCCACCACTTTCTGATA-3′ (GenBank accession no. NM_000518.4). The cDNA was directionally cloned into the pcDNA3.1-myc plasmid (Invitrogen, Carlsbad, CA, USA) and the orientation was verified by sequencing.

### MES23.5 dopaminergic cell cultures

Mesencephalon × neuroblastoma N18TG2 hybrid (MES23.5 dopaminergic) cells were a gift from Dr. Weidong Le at Baylor College of Medicine [38]. These were cultured in Dulbecco's Modified Eagle's Medium/F12 (Gibco, Grand Island, NY, USA) supplemented with 5% fetal calf serum and Sato's ingredients and transfected with plasmids using Lipofectamine 2000 (Invitrogen, Carlsbad, CA, USA) according to the manufacturer's instructions. Briefly, 1 day before transfection, cells were seeded at 1 × 10^7^ per flask, which were precoated with poly-l-lysine [39]. Plasmids pcDNA3.1-myc (myc) and pcDNA3.1-myc-nHb (nHb/myc) (9 μg) were mixed with 20 μl Lipofectamine 2000 in Opti-MEM (Gibco). Cells were incubated at 37°C in a CO_2_ incubator, and protein expression was verified by western blotting and immunocytochemistry.

### Animals

Cynomolgus monkeys (*Macaca fascicularis*; *n* = 12) were purchased from a local nonhuman primate breeder (Grandforest Co., Guangxi, China) with detailed individual birth records and quarantine certificates. All animals were healthy and without physical impairments. The animals were acclimated to the laboratory environment for at least 2 months before dissection and were divided into three age groups: young (range: 3-4 years; *n* = 4), middle age (range: 10-12 years; *n* = 4), and old (range: ≥ 15 years; *n* = 4). Animals were housed in a primate facility (Wincon TheraCells Biotechnologies Co., Nanning, China) accredited by the Association for Assessment and Accreditation of Laboratory Animal Care under a 12:12-h light/dark cycle with free access to an uninterrupted reverse osmosis water supply. Food was available twice daily and supplemented with fresh fruit and vegetables. The experimental protocol was approved by the Institutional Animal Care and Use Committee of Wincon TheraCells Biotechnologies (permit no. WD-0312010). All animal experiments were carried out in accordance with the National Institutes of Health (NIH) Guide for the Care and Use of Laboratory Animals (NIH publication no. 85-23, revised 1996), and were approved by the local animal care and use committee.

### Dissection of monkey brain tissue

Under deep anesthesia, the thoracic cavity of each monkey was opened, and the heart was exposed. The animal was perfused from the aorta with 2000-3000 ml of 0.01 mM phosphate-buffered saline (PBS) (pH 7.4). The brain was removed from the skull, and the brain tissue was dissected on ice, snap-frozen in liquid nitrogen, and stored at −80°C until use.

### Isolation of subcellular fractions

Mitochondrial, cytosolic, and membrane fractions were isolated from brain tissue or cell cultures according to a previously described protocol [8], with slight modifications. Briefly, brain tissue samples or cells were homogenized in mitochondrial isolation buffer containing 0.32 M sucrose, 1 mM EDTA, and 10 mM Tris-HCl (pH 7.4). Cytosolic, crude mitochondrial, and membrane fractions were obtained by differential centrifugation. Mitochondria were isolated from crude mitochondrial fractions with Percoll (Bioshop, Burlington, Canada) by density gradient centrifugation. Protein concentration was determined using the Bicinchoninic Acid Protein Assay kit (Thermo Fisher Scientific, Waltham, MA, USA) according to the manufacturer's instructions.

### Enzyme-linked immunosorbent assay (ELISA) for Hb-α-syn complex

Hb-α-syn complex levels in brain tissue, cell lysates, and RBCs were measured by ELISA using a mouse monoclonal anti-Hb antibody (ab77125; Abcam, Cambridge, MA, USA) and biotinylated mouse monoclonal anti-α-syn (3D5) antibody [40] for capture and detection, respectively. After completion of the immunoreaction, samples were incubated with 100 μl ExtrAvidin alkaline phosphatase (E-2636; Sigma-Aldrich, St. Louis, MO, USA) diluted 1:20,000 in blocking buffer followed by the enzyme substrate p-nitrophenyl phosphate (N1891; Sigma-Aldrich). The reaction was allowed to proceed for 30 min at room temperature, after which the absorbance was read at 405 nm using a Multiskan MK3 microplate reader (Thermo Scientific, UT, USA).

### Measurement of cell viability

To assess cell viability, MES23.5 cells seeded at a density of 1 × 10^4^/well in a 96-well plate were transfected with myc or nHb/myc plasmid; 24 h later, cells were treated with α-syn (10 μM) for 1, 3, or 6 h or left untreated. Cell viability was estimated with the 3-(4, 5-dimethylthiazol- 2-yl)-2,5-diphenylte-trazolium bromide assay [41].

### Western blot analysis

Western blotting was performed as previously described [41]. Briefly, protein samples (20 μg protein/lane) were separated by 12.5% sodium dodecyl sulfate polyacrylamide gel electrophoresis (SDS-PAGE) and transferred to a polyvinylidene difluoride membrane. Blots were separately probed with mouse monoclonal 3D5 anti-α-syn (1: 5000) and anti-Hb (1: 1000; ab77125, Abcam, MA, USA) primary antibodies, as well as rabbit polyclonal anti-actin (1: 1000; sc-7210, Santa Cruz Biotechnology, Santa Cruz, CA, USA), mouse monoclonal anti-voltage-dependent anion channel (1: 500; ab14734, Abcam, MA, USA), and rabbit polyclonal anti-calnexin (1: 1000; 2433s, Cell Signaling Technology, Danvers, MA, USA) antibodies as loading controls for cytosolic, mitochondrial, and membrane fractions, respectively. The blots were probed with appropriate secondary antibodies conjugated with horseradish peroxidase (1:5000; Vector Laboratories, Burlingame, CA, USA). Immunoreactivity was detected using enhanced chemiluminescence reagent (Promega, Madison, WI, USA).

### Immunoprecipitation (IP)

Extracts (100 μg) from MES23.5 cells or tissue from the monkey striatum or cerebellum were incubated overnight at 4°C with mouse monoclonal anti-Hb (3 μg) antibody under constant rotation. Protein/antibody mixtures were then incubated for 1 h at 4°C with protein G-sepharose beads in IP buffer composed of 10 mM Tris-Cl (pH 7.5), 150 mM NaCl, 2 mM EDTA, and 0.5% Triton X-100. The beads were collected by centrifugation at 10,000 × *g* for 1 min and washed three times with IP buffer to remove nonspecifically bound proteins. The beads were resuspended in SDS-PAGE loading buffer (60 μl/tube) and heated at 95°C for 5 min, and then removed by centrifugation at 10,000 × *g* for 1 min. The supernatant was analyzed by western blotting.

### Immunocytochemistry and confocal microscopy

MES23.5 cells were transfected with myc or nHb/myc plasmid for 24 h with or without α-syn treatment, then fixed with 3.7% paraformaldehyde and permeabilized with 0.2% Triton X-100. Cells were then incubated overnight at 4°C with a mouse monoclonal anti-myc (1:1000; 631206, Clontech, Mountain View, CA, USA) or rabbit polyclonal anti-α-syn (1:5000) antibody, followed by incubation for 1 h with Alexa Fluor 488-conjugated goat anti-mouse (1:500, CA11001s, Invitrogen, Carlsbad, CA, USA) or Alexa Fluor 594-conjugated rabbit (1:500, CA11012s, Invitrogen, Carlsbad, CA, USA) IgG. Nuclei were counterstained with 4′,6-diamidino-2-phenylindole (DAPI; Invitrogen, Carlsbad, CA, USA) and cells were visualized under a confocal microscope (DM4000B; Leica, Wetzlar, Germany).

### Terminal deoxynucleotidyl transferase (TdT)-mediated dUTP nick end labeling (TUNEL)

Apoptosis was detected with the TUNEL assay using the *In Situ* Cell Death Detection kit (Roche, Mannheim, Germany). Briefly, MES23.5 cells were seeded on poly-l-lysine-coated glass cover slips, grown to 60% confluence, transfected, and treated with α-syn or vehicle. The cells were then fixed with 4% paraformaldehyde, rinsed in equilibration buffer and incubated with nucleotide mix and terminal deoxynucleotide transferase enzyme. The reaction was halted by adding the saline-sodium citrate buffer. Cells were stained with DAPI, and images were acquired with a confocal microscope (Leica DM4000B, Wetzlar, Germany).

### JC-1 staining for detection of mitochondrial membrane potential

Cells were stained with JC-1, a mitochondrial membrane potential sensor (Biotium, Hayward, CA, USA), and sorted by flow cytometry. A 1-ml volume of cell suspension containing 1 × 10^6^ cells was centrifuged at 400 × *g* for 10 min. The supernatant was discarded, and the pellet was resuspended in 0.5 ml JC-1 staining solution [10 μg/ml] and incubated at 37°C for 15 min, then centrifuged at 400 × *g* for 10 min. The pellet was resuspended and washed with PBS; the centrifugation-resuspension steps were repeated three times. JC-1 fluorescence was detected on a FACSAria flow cytometer (Becton Dickinson, Franklin Lakes, NJ, USA).

### Statistical analysis

Data are expressed as mean ± standard deviation (SD). Differences between groups were evaluated by two-way analysis of variance followed by Tukey's multiple comparisons test. Pearson correlation analysis was used to assess the association between nHb-α-syn levels in cytosolic, mitochondrial, and membrane fractions and pHb-α-syn in RBCs. *P* < 0.05 was considered statistically significant.
